# Tyrosine Hydroxylase and DOPA Decarboxylase Are Associated With Pupal Melanization During Larval–Pupal Transformation in *Antheraea pernyi*

**DOI:** 10.3389/fphys.2022.832730

**Published:** 2022-04-07

**Authors:** Qi Wang, Liang Zhong, Yong Wang, Shengwei Zheng, Yumeng Bian, Junhao Du, Ruisheng Yang, Wei Liu, Li Qin

**Affiliations:** ^1^College of Bioscience and Biotechnology, Insect Resource Research Center for Engineering and Technology of Liaoning Province, Shenyang Agricultural University, Shenyang, China; ^2^Sericultural Research Institute of Liaoning Province, Fengcheng, China

**Keywords:** *Antheraea pernyi*, pupal melanization, tyrosine hydroxylase, DOPA decarboxylase, RNA interference, enzyme inhibitor

## Abstract

In insects, melanism plays important roles in defense, immunoreactions, and body color. The underlying molecular mechanisms of melanism in different insects are diverse and remain elusive. In contrast to another silkworm, *Bombyx mori*, the Chinese oak silkworm, *Antheraea pernyi*, produces melanic pupae under natural environmental conditions. DOPA and dopamine synthesis are crucial for melanin formation. Disruption of these processes reportedly influences body colors in many insects. Most research focuses on newly emerged pupae, and the larval process preceding pupation remains unknown. Due to the large size and long pupation period in *A. pernyi*, the entire process was studied at least every 12 h. The expression patterns of tyrosine hydroxylase (*TH*) and DOPA decarboxylase (*DDC*), which are involved in DOPA and dopamine synthesis in the epidermis, were evaluated during larval–pupal metamorphosis. We also performed RNA interference (RNAi) and used enzyme inhibitors to examine morphological changes. The amino acid sequences of TH and DDC share 90.91% and 86.64% identity with those of *B. mori*. *TH* and *DDC* expression was upregulated during the 48–72 h period prior to pupal emergence, especially at 60 h. RNAi of *TH* and *DDC* induced partial melanism in some pupae. The inhibitors 3-iodo-tyrosine (3-IT) and L-α-methyl-DOPA (L-DOPA) influenced pupal melanization. Different concentrations of inhibitors led to pupal deformity and even mortality. Four different monoamines, only DOPA and Dopamine synthezed from Tyrosine will be influenced by TH and DDC inhibitor. These results indicate that *TH* and *DDC* are key genes associated with pupal melanization during larval–pupal transformation in *A. pernyi.* Overall, our results suggest that *TH* and *DDC* expression alterations in a particular stage can affect body color, setting the molecular basis for artificial control of pupal melanization.

## Introduction

In insects, body pigments are mainly synthesized in epidermal cells through a complex cascade of biochemical reactions. Body pigments are important for insect morphology and protection insects from exogenous physical injury (Andersen, 2010). Melanism, caused by the over-deposition of the pigment melanin in several insect species, is a conspicuous, dark-colored phenotypic variation reflected in external morphology. Melanin is involved in cuticle sclerotization, color patterning, clot formation, melanotic encapsulation, organogenesis, and innate immunity (Whitten and Coates, 2017). The melanic phenotype may only be visible during one or two stages in holometabolous insects, such as larval (*Manduca sexta*, *Spodoptera littoralis*, and *Bicyclus anynana*), pupal (*Spodoptera exigua* and *Biston betularia*), adult (*Araschnia levana* and *Mythimna separata*), larval and adult (*Bombyx mori mln* mutant strain), larval and pupal (*B. mori* sooty mutant strain), or pupal and adult stages (*Helicoverpa armigera*) (Liu et al., 2015a).

Unlike in vertebrates, melanism has different mechanisms in insects owing to the diverse cellular and developmental bases of insect pigmentation. Although certain melanic mutant species (*Papilio glaucus*, *B. mori*, *S. exigua*, and *M. sexta*) have been used to investigate the molecular mechanisms underlying melanism, the genetic mechanisms causing this diversity in insects of different stages remain unclear (Liu et al., 2015a). For instance, in a black larval mutant of *M. sexta*, decarboxylase (DDC) expression was detected at the end of larval molting (Hiruma and Riddiford, 2009). In a mutant of *B. mori*, some level of melanization occurred due to the deletion of certain genes (N-β-alanyl dopamine synthase gene, *ebony*, and arylalkylamine-N-acetyltransferase gene, *AANAT*) (Futahashi et al., 2008; Dai et al., 2010). Tyrosine hydroxylase (TH) catalyzes tyrosine conversion into DOPA (L-dihydroxyphenylalanine), a precursor of melanization (Christensen et al., 2005). Compared with wild-type pupae, a melanic mutant strain of *S. exigua* showed no difference in gene sequences. Both TH and DDC were considerably overexpressed in the mutant strain in late-pre-pupa and 0-h pupa (Liu et al., 2015a). These findings indicate that TH and DDC are involved in insect melanization.

Melanin synthesis is a complex biochemical process involving the oxidation of DOPA or dopamine. TH participates in the first step of DOPA production from tyrosine (Futahashi and Fujiwara, 2005; Gorman et al., 2007). DOPA can be further converted to DOPA melanin, but this progression is still unclear. To the best of our knowledge, there are no reports on the involvement of *TH* and *DDC* during pupal metamorphosis in the edible silkworm pupa *Antheraea pernyi*.

The Chinese oak silkworm (*A. pernyi*) (Guérin-Méneville) is a widely reared a lepidopteran in China. *A. pernyi* is used for silk production and is also an edible insect (Li et al., 2020). The pupae are available on the market almost throughout the year, as they are artificially cultivated using pupal diapause. The annual production of cocoons has been reported to be approximately 7.10–9.38 × 10^4^ t and North China, including Liaoning, Jilin, Heilongjiang, and Shandong provinces, accounts for roughly 70% of pupae consumed (Zhu et al., 2017).

In this study, to understand the whole process of larval–pupal transformation in edible silkworm *A. pernyi*, we monitored the morphological changes and melanin formation every 12 h (pre-pupae) or 3 h (newly pupated pupae). We aimed to identify the developmental stage in which *TH* and *DDC*, two key genes responsible for melanin precursors, induce enzyme synthesis for melanization. We analyzed the expression patterns using quantitative reverse transcription-PCR (RT-qPCR) of two key genes. We performed RNA interference (RNAi) and used enzyme inhibitors influencing morphological variations. Overall, our results suggest that TH and DDC are associated with pupal melanization during larval–pupal metamorphosis in *A. pernyi*. To the best of our knowledge, this is the first report on the pupal melanization process in Saturniidae.

## Materials and Methods

### Silkworm

The bivoltine strain Xuanda No. 1 was reared in the Research Institute of Tussah, Shenyang Agricultural University (Shenyang, Liaoning province, China). The larvae were fed oak tree leaves (*Quercus wutaishanica*) until they started spinning cocoons by the end of the fifth instar larval stage. Some individuals were chosen for the experiment on the day when they started spinning cocoons. The larvae were placed in an incubator at 23°C and 60 ± 10% relative humidity. Considering that the development of individuals was asynchronous, 2,000 larvae were selected to ensure several individuals were in the same developmental stage (especially the pre-pupa stage). Tissues including the brain, gonad, midgut, Malpighian tubule, silk gland, tracheae, fat body, hemolymph, and especially the epidermis, were dissected every 12 h from pre-pupae and every 3 h after pupal emergence. All samples were frozen at −80°C for subsequent use.

### Sampling During Metamorphosis From Pre-pupa to Pupa

After cocoon formation, shrunk larvae were observed by dissecting a piece of each cocoon. Larvae of the same stage were placed in an incubator at 23°C. Samples were collected every 12 h before pupal emergence. At 12 h after pupal emergence, samples were dissected every 3 h since the epidermis changes quickly. After an additional 12 h, tanning black pupae showed complete metamorphosis. Pupal morphological changes were recorded using a camera (Canon 5D Mark III, Tokyo, Japan).

### Cloning of Tyrosine Hydroxylase and DOPA Decarboxylase in *Antheraea pernyi*

The total RNA was isolated from the epidermis using the RNAiso Plus kit (Takara, Dalian, China) and quantified by spectrophotometry (Biodrop uLITE, Biochrom, United Kingdom). The cDNA was synthesized with a PrimeScript™ One Step RT-PCR kit (Takara, Dalian, China). Primers for cloning were designed from an epidermis transcriptome library constructed in-house using Primer Premier version 6.0 software (Table 1). Amplification was performed on an S1000™ Thermal Cycler (Bio-Rad, Hercules, CA, United States) under an initial 3 min denaturation at 94°C, followed by 34 cycles of 30 s denaturation at 94°C, 30 s annealing at 55°C, 45 s extension at 72°C, and a final extension at 72°C for 5 min. Polymerase chain reaction (PCR) products were electrophoresed on a 1.2% agarose gel and purified using an EasyPure^®^ Quick Gel Extraction kit (Transgen, Beijing, China). The products were cloned into the pMD18-T vector (Takara, Dalian, China) for sequencing (Sangon Biotech Co., Ltd., Shanghai, China) using PCR to confirm positive clones.

**TABLE 1:** Primer sequences used in gene cloning, RT-PCR, qRT-PCR and RNAi.

Name	Primers
*TH*	Forward: CTCGTTCAGGCTCTACCAATCC
	Reverse: GTGCTCCATTTTTTATTCATTTATG
*DDC*	Forward: TGGAGGTCGGAGACTTCAAAGAGTT
	Reverse: TGCCATCACTGCTGTCCAAGGT
Sq*-TH*	Forward: CCTGGTTTTGCCGACAGGGAGTA
	Reverse: CGGTAGGTTTACGTAAAAAGTTG
Sq*-DDC*	Forward: TCGCCGAAGTTCCACGCCTAT
	Reverse: CCTGCTCGCTCCACTGAAGAATG
Sq*-β-actin*	Forward: CCAAAGGCCAACAGAGAGAAGA
	Reverse: CAAGAATGAGGGCTGGAAGAGA
qPCR-*TH*	Forward: TGTGGCAACTTTTTACGC
	Reverse: TCAGGGGTATGGAATGGTG
qPCR*-DDC*	Forward: GCAACATTGGTGGCACTTCTTGG
	Reverse: GTCCTGCTCGCTCCACTGAAGA
qPCR*-actin*	Forward: ACCAACTGGGACGACATGGAGAAA
	Reverse: TCTCTCTGTTGGCCTTTGGGTTGA
RNAi*-TH*	Forward: TAATACGACTCACTATAGGGCGCTAACCGAGGA GGAAGTA
	Reverse: TAATACGACTCACTATAGGGTGGGATGGCATCA CCGTATT
RNAi*-DDC*	Forward: TAATACGACTCACTATAGGGGGCAAGTAGTACC GTCTGTG
	Reverse: TAATACGACTCACTATAGGGGCTCGCTCCAC TGAAGAATG
RNAi*-EGFP*	Forward: TAATACGACTCACTATAGGGAGATAAACGGCCA CAAGTTCAGC
	Reverse: TAATACGACTCACTATAGGGAGAGTGTTCTGCT GGTAGTGGTC

### Amino Acid Sequence Alignment and Bioinformatic Analysis

Amino acid sequences of *TH* and *DDC* were deduced based on the open reading frame (ORF) sequences. ExPASy^1^ was used to determine the theoretical isoelectric point, molecular weight (Mw), and SignalP. Conserved domains of TH and DDC were predicted using the National Center for Biotechnology Information^2^ tools. A neighbor-joining (NJ) phylogenetic tree was generated using MEGA7.0 and Clustal X (1000 bootstrap replicates) (Larkin et al., 2007; Tamura et al., 2011). The tree was displayed under iTOL version 6, a web-based tool^3^ (Ivica and Peer, 2016). *Cis*-element were analyzed using 2000 bp sequence before ORF in genome sequence by *Cis*-Acting Regulatory Element-PlantCARE^4^ (Lescot et al., 2002).

### Tyrosine Hydroxylase and DOPA Decarboxylase Tissue Distribution

During the larval–pupal stage, semi-quantitative PCR (sqPCR) was performed to record the accumulation of *TH* and *DDC* mRNAs in indicated tissues. β*-actin* (GenBank accession number: GU176616) was used as a reference gene and the respective primers are listed in Table 1. The cDNA was synthesized according to the protocol of the Reverse Transcription System kit (Promega, Madison, WI, United States). One microliter of the first-strand cDNA was used as the template in 25 μL of PCR mixtures on an S1000™ Thermal Cycler. The PCR was performed under the following conditions: initial denaturation at 94°C for 3 min, 28 cycles of denaturation at 94°C for 30 s, annealing at 55°C for 30 s, extension at 72°C for 20 s, and a final extension at 72°C for 5 min. A parallel experiment with no template was run simultaneously. Ten microliters of the PCR products were visualized with 4S GelRed (Sangon, Shanghai, China) after electrophoresis. All samples were analyzed in triplicates. The PCR products were sequenced by Sangon Biotech Co., Ltd. (Shanghai, China) to verify the accuracy of amplicons.

### RT-qPCR Analysis

RT-qPCR was performed to detect gene expression levels, using the stably expressed β*-actin* (Sun et al., 2017) as the reference gene (Bustin et al., 2009). Primers used in RT-qPCR profiling are presented in Table 1. RT-qPCR was performed using SYBR^®^ Premix Ex Taq™II kit (TaKaRa, Shiga, Japan) and a LightCycler^®^ 480 system (Roche Diagnostics, Mannheim, Germany). The qPCR mixture and the thermal cycling conditions were set according to a previous study (Wang et al., 2021). Approximately 3 days before pupal emergence and 1 day after pupal melanization, the epidermis was dissected for RT-qPCR. The experiment included three independent biological replicates for each time point. The comparative C_*T*_ (ΔΔC_*T*_) method was used.

### RNA Interference of Tyrosine Hydroxylase and DOPA Decarboxylase

Approximately 500-bp templates, for preparing double-stranded RNA, were amplified by PCR using the primers listed in Table 1. The ds*TH* and ds*DDC* fragments were synthesized using the HiScribe T7 Quick High Yield RNA Synthesis Kit (New England Biolabs, Ipswich, United States). The dsRNA was diluted to 2 μg/μL and 30 μg was injected into the dorsal abdomen of each pupa. The dsRNA for *EGFP* was synthesized and injected as a negative control. The injection time, 36 h, was selected based on *TH* and *DDC* expression patterns. To analyze the knockdown level of *TH* and *DDC* transcripts after RNAi, gene expression was evaluated by RT-qPCR at 24 h post-injection. Three individuals were selected for the morphological analysis of each group.

### Morphological Variations and Malformation Rate After Enzyme Inhibitor Injection

The TH and DDC inhibitor, 3-iodo-tyrosine (3-IT), and _*L*_-α-methyl-DOPA (_*L*_-DOPA) (Sigma) were diluted in a DMSO solution. The concentrations were 50, 100, and 200 mmol/L. Each pupa was administered 30 μL of the inhibitors at different concentrations. An equal volume of DMSO was injected into pupae in the same stage as the negative control (approximately 36 h before pupal emergence). There were 30 pupae in each group and the numbers of malformed and dead pupae were recorded. The pupal morphological variations in the epidermis color were compared among the 0, 50, and 100 mmol/L 3-IT and L-DOPA groups.

### Four Monoamines Content Variations After Enzyme Inhibitor Injection

After injected with 30 μL 100 mmol/L 3-IT and L-DOPA (approximately 36 h after sample collection), the epidermis were dissected for monoamines test after 36 h (a few hours before pupal emergence). Each pupa epidermis were disrupted by liquid nitrogen grinding. For DOPA, Dopamine and Norepinephrine testing, each 0.1 g tissues were added into 1 mol/L perchloric acid and then for ice-bath ultrasonic extraction. After centrifugation at 12,000 rpm for 10 min, the supernatant was extracted using 0.22 μm microfiltration membrane for LC-MS/MS analysis by Shimadzu LC-20AT HPLC system (Shimadzu, Kyoto, Japan). A Ulimate column (150 mm × 4.6 mm) packed with Lichrosorb reversed phase C18 (5 μm) was used. Ten microliter sample was eluted with methanol (A) and sodium acetate (B) for gradient elution at a flow rate of 1 mL/min at 30°C.

For Octopamine testing, 2 mL 5% TCA was added. After centrifuge 10 min at 12,000 rpm, 100 μL supernatant was added into 500 μL Na_2_CO_3_–NaHCO_3_ buffer solution and then 2 mL 10 mg/mL dansyl chloride-acetone solution for 15 min in dark. The supernatant was extract by 1 mL ethyl acetate and dried in nitrogen, then resuspended in 1 mL acetonitrile. Solutions were purified by 0.22 μm microfiltration membrane for LC-MS/MS analysis. Using Wufeng LC-100 HPLC system (Wufeng, Shanghai, China), a Ulimate column (150 mm × 4.6 mm) packed with Lichrosorb reversed phase C18 (5 μm) was used. Twenty microliter sample was eluted with 90% acetonitrile and 10% 0.1 mol/L ammonium acetate (A) and 10% acetonitrilesodium and 90% 0.1 mol/L ammonium acetate (B) for gradient elution at a flow rate of 0.8 mL/min at 35°C.

### Statistical Analyses

Statistical analyses were performed using SPSS version 21.0 software (IBM, Chicago, IL, United States). Relative expression levels were compared using Student’s *t*-tests. The results of three biological replicates were presented as the mean ± standard deviation. Significant differences were determined using Duncan’s multiple range tests (*p* < 0.05). The graphs were plotted using GraphPad Prism 6 (GraphPad, San Diego, CA, United States).

## Results

### Morphological Changes During Metamorphosis From Pre-pupa to Pupa

**FIGURE 1:**
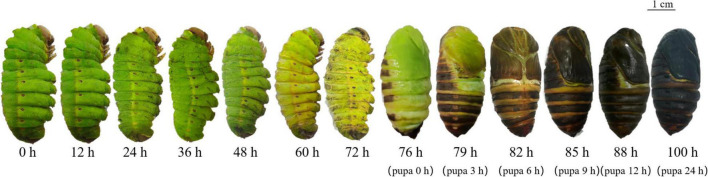
Morphological changes from the larval to pupal stages. Changes 3 days before and 1 day after pupal emergence were recorded. Three days before pupal emergence was considered 0 h and 76 h was the beginning of pupal emergence.

After cocoon spinning, most individuals metamorphosized from larvae to pupae. Some individuals did not complete this process due to disease, wounds, or abnormal hormone secretion. *A. pernyi* metamorphosis, from cocoon to pupa, required 10–14 days. During this transformation, the larvae underwent morphological changes and showed typical gene expression patterns. Cuticles exuviated 4 h before pupal emergence and breaking from old cuticle process consistently lasted 10 min. When pupae emerged from the old cuticle, the soft skin turned black and started to tan. Cuticles underwent melanization just after pupal emergence, and half of the cuticle turned black within 3 h. The epidermis was completely melanized within 9 h and almost completely black and tanned within 24 h (Figure 1).

### Gene Cloning and Sequence Analysis of Tyrosine Hydroxylase and DOPA Decarboxylase in *Antheraea pernyi*

Tyrosine hydroxylase and DOPA decarboxylase contain a 1686- and 1440-bp ORF, respectively. *TH* encodes a putative protein of 561 amino acids, 63.5 kDa MW, with an isoelectric point of 5.29. *DDC* encodes a putative protein of 479 amino acids, 54.2 kDa MW, with a PI of 5.82. The two genes were named *ApTH* and *ApDDC* (GenBank accession numbers MW677189 and MW677190). *ApTH* has seven exons and six introns in the protein-coding region of the 6984-bp DNA sequence (Supplementary Figure 1), whereas *ApDDC* has four exons and three introns in its 9784-bp DNA sequence (Supplementary Figure 2). Bioinformatic analyses revealed that both ApTH and ApDDC proteins included no signal peptide. Both *ApTH* and *ApDDC* found many *cis*-element, including CAAT-box (Supplementary Figure 3).

### Sequence Homology Alignment and Phylogenetic Analysis

**FIGURE 2:**
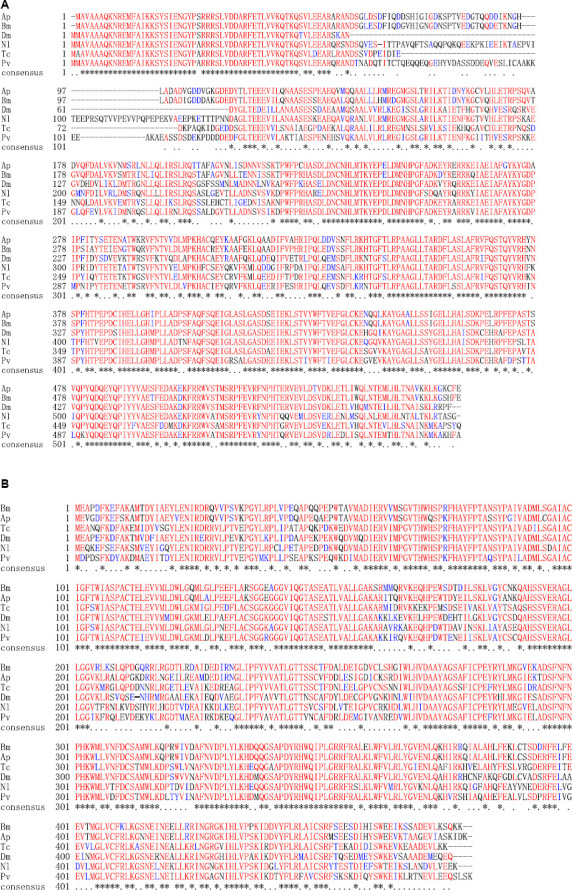
Alignment of deduced amino acid sequences of TH **(A)** and DDC **(B)**. Amino acids with identical and similar residues are marked with asterisks and dots. Species abbreviations and accession nos. of the sequences used in the alignment are BmTH*-Bombyx mori* (NP_001138794.1), PvTH*-Polyrhachis vicina* (AEC14314.1), TcTH*-Tribolium castaneum* (NP_001092299.1), NlTH*-Nilaparvata lugens* (QEE04288.1), DmTH*-Drosophila melanogaster* (CAA53802.1), ApTH-*Antheraea pernyi* (QZU75311.1); BmDDC*-B. mori* (AAK48988.1), TcDDC*-T. castaneum* (ABU25222.1), DmDDC-*D. melanogaster* (NP_523600.5), NlDDC*-N. lugens* (QEE04289.1), PvDDC*-P. vicina* (AFI80897.1), ApDDC-*A. pernyi* (QZU75312.1).

**FIGURE 3:**
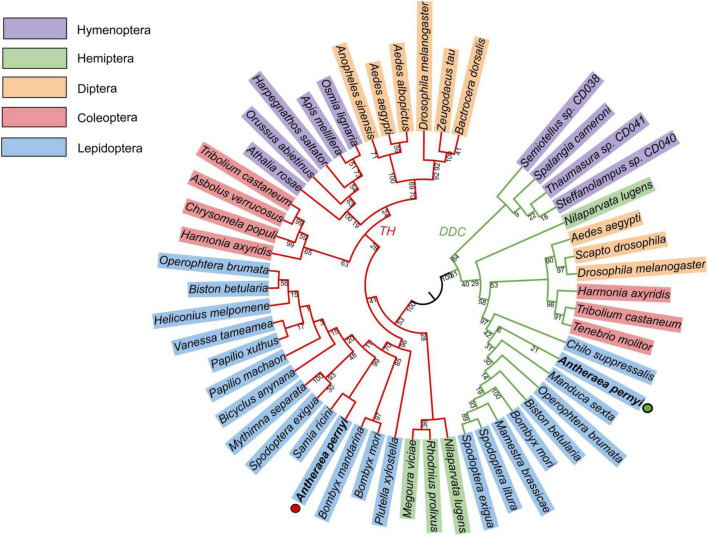
Phylogenetic tree constructed using TH and DDC sequences of different insect species with MEGA7.0. Bootstrap percentage values are marked on the red (TH) and green (DDC) branches. GenBank accession numbers are shown as follows. TH: *Bactrocera dorsalis* (AVP73886.1); *Zeugodacus tau* (QBE90477.1); *Drosophila melanogaster* (CAA53802.1); *Aedes albopictus* (XP_019527163.2); *Aedes aegypti* (XP_021696291.1); *Anopheles sinensis* (AMZ03511.1); *Apis mellifera* (NP_001011633.1); *Harpegnathos saltator* (EFN85710.1); *Orussus abietinus* (XP_012279464.1); *Athalia rosae* (XP_012269076.1); *Tribolium castaneum* (NP_001092299.1); *Asbolus verrucosus* (RZC40828.1); *Chrysomela populi* (AWK23447.1); *Harmonia axyridis* (QER78576.1); *Operophtera brumata* (KOB71174.1); *Biston betularia* (ADF43213.1); *Heliconius melpomene malleti* (ADU32895.1); *Vanessa tameamea* (XP_026500806.1); *Papilio machaon* (NP_001303944.1); *Papilio xuthus* (NP_001299711.1); *Bicyclus anynana* (AGT62463.1); *Mythimna separata* (BAF32573.1); *Spodoptera exigua* (AFG25778.1)*; Samia ricini* (BAF64534.1); *Bombyx mori* (NP_001138794.1); *Bombyx mandarina* (ADV56709.1); ApTH-*Antheraea pernyi* (QZU75311.1); *Plutella xylostella* (NP_001292449.1); *Megoura viciae* (ASU09659.1); *Rhodnius prolixus* (ANZ03350.1); *Nilaparvata lugens* (QEE04288.1); *Osmia lignaria* (XP_034181574.1). DDC: *Drosophila melanogaster* (NP_523600.5); *Aedes aegypti* (AAC31639.1); *Tribolium castaneum* (ABU25222.1); *Harmonia axyridis* (AMQ13055.1); *Operophtera brumata* (KOB76562.1); *Biston betularia* (ADF43201.1); *Spodoptera exigua* (AFG25780.1); *Spodoptera litura* (AHB23865.1); *Bombyx mori* (AAK48988.1); ApDDC-*A. pernyi* (QZU75312.1); *Nilaparvata lugens* (QEE04289.1); *Mamestra brassicae* (BAB68545.1); *Manduca sexta* (QDR50989.1); *Chilo suppressalis* (AKL78850.1); *Tenebrio molitor* (BAA95568.1); *Scapto drosophila* (AAC67585.1); *Steffanolampus* sp. CD040 (ABJ90384.1); *Thaumasura* sp. CD041 (ABJ90385.1); *Spalangia cameroni* (ABJ90383.1); *Semiotellus* sp. CD038 (ABJ90382.1).

Homology analysis revealed that the predicted ApTH amino acid sequence (QZU75311.1) had a sequence identity of 90.91% with the TH protein of *Bombyx mori* (NP_001138794.1) and 71.00% with that of *Tribolium castaneum* (NP_001092299.1). According to multiple alignments (Figure 2A), TH sequences from five insects (including Lepidoptera *A. pernyi* and *B. mori*, Diptera *Polyrhachis vicina*, Coleoptera *T. castaneum*, and Hemiptera *Nilaparvata lugens*) were conserved in the N-terminal, including several regions (230–287, 350–448, and 487–537 aa). The homology analysis showed that ApDDC (QZU75312.1) had a sequence identity of 86.64% and 73.07% with BmDDC (AAK48988.1) and TcDDC (ABU25222.1), respectively. The ApDDC had a sequence identity of 80.94% with the other three species (*Drosophila melanogaster*, *N. lugens*, *P. vicina*) (Figure 2B). The conserved sequences were located at 58–159 and 270–370 aa. The five groups of selected insects were separated in the NJ phylogenetic tree, indicating TH and DDC segregate into two groups. Based on DDC sequences, TH of *A. pernyi* has a close relationship with TH of *Samia ricini* and *A. pernyi* showed a close genetic relationship with *M. sexta* (Figure 3).

### Tissue-Specific Expression Profiles of *ApTH* and *ApDDC*

**FIGURE 4:**
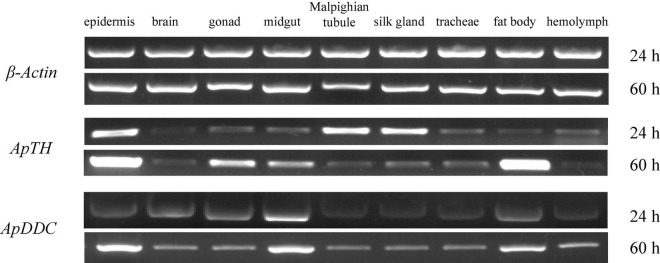
Relative expression of *ApTH* and *ApDDC* in tissues was quantified by semi-quantitative RT-PCR. The reference gene was β*-actin*, two different time (24 h and 60 h) were selected for PCR amplification in tissues.

The *ApTH* transcripts were detectable in all tissues, especially the epidermis and hemolymph at 24 h. The epidermis and fat body incresed higher than other tissues at 60 h. *ApTH* shows down regulated at 60 h in Malpighian tubule and silk gland. At 24 h, *ApDDC* expression was higher in the midgut, epidermis, brain, gonads, and fat body than in Malpighian tubule, silk gland, tracheae, and hemolymph tissues. High content of *ApDDC* was tested in epidermis at 60 h (Figure 4).

### Expression Profiles of *ApTH* and *ApDDC* From Pre-pupa to Pupa

**FIGURE 5:**
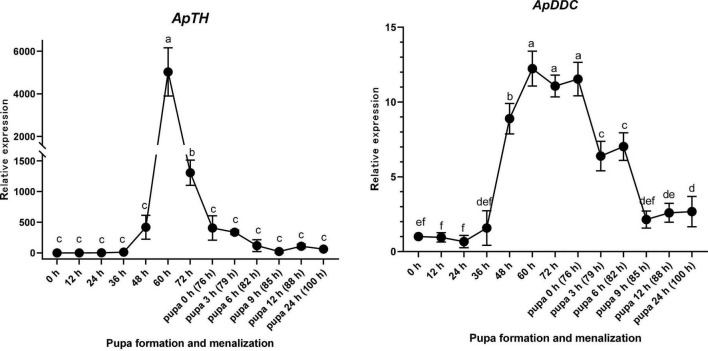
Relative expression levels of *ApTH* and *ApDDC* in the epidermis of *Antheraea pernyi* during the larva–pupa stage, determined by qPCR. Three replicates were considered; the bar represents mean ± SD (*n* = 3), and bars annotated with the same lower-case letters are not significant difference by Duncan’s multiple range test (*p* < 0.05).

*ApTH* was expressed from 36 h after sample collection (1 day before pupal emergence). Compared with the beginning of sampling, *ApTH* showed a marginal (419-fold) increase at 48 h and then a substantial increase in the next 12 h, peaking at 60 h (5,028-fold higher than that at 0 h). Subsequently, *ApTH* expression consistently reduced 60 h after sampling commenced. At 76 h (0 h of pupal emergence), expression decreased to the same level as observed at 48 h and remained low for the next 24 h. From 36 to 60 h, *ApDDC* expression increased quickly and peaked (12.23-fold). *ApDDC* expression remained high until 76 h (pupal emergence) and 9 h later, after pupal emergence (85 h), decreased by 5.39-fold compared with the beginning of pupal emergence (76 h). The expression of both *ApTH* and *ApDDC* was highest at 60 h (Figure 5).

### RNAi of *ApTH* and *ApDDC*

**FIGURE 6:**
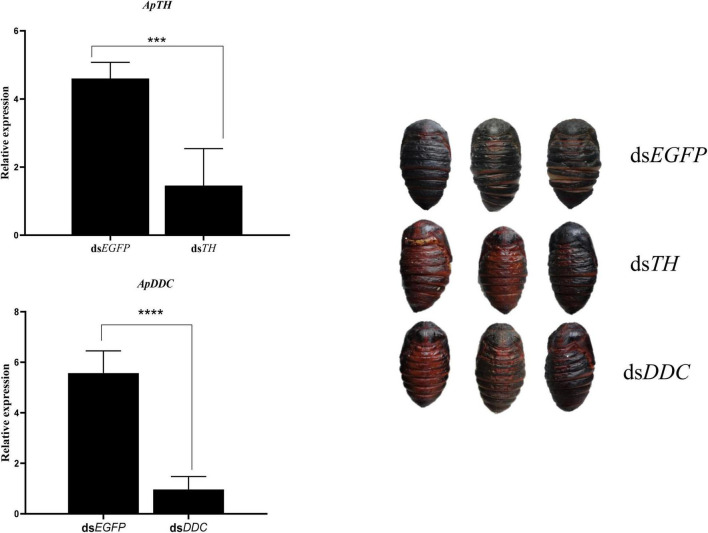
Effects of knockdown of *ApTH* and *ApDDC* during the prepupal stage and the corresponding morphological changes. A significant difference was observed using Student’s t test. *** *p* < 0.001, **** *p* < 0.0001.

After injecting ds*TH* and ds*DDC* into the pre-pupa at 36 h, RT-qPCR of samples collected at 60 h was performed to test target gene expression. *ApTH* and *ApDDC* expression was considerably suppressed by dsRNA of the target genes compared with that in the ds*EGFP* group. *ApTH* expression decreased by 68.3%, compared with ds*EGFP* expression (*p* < 0.01). The *ApDDC* transcript level significantly decreased by 82.7%, compared to the control. In the morphological analysis, two pupae in the ds*TH* group exhibited negligible melanin pigmentation in backside of the abdomen. In contrast, only one pupa in the ds*DDC* group showed obvious brown coloration, especially in the middle back epidermis. In the ds*EGFP*-injected control group, all pupae showed melanization after 24 h (Figure 6).

### Enzyme Inhibitor Influenced Malformation and Melanization

**FIGURE 7:**
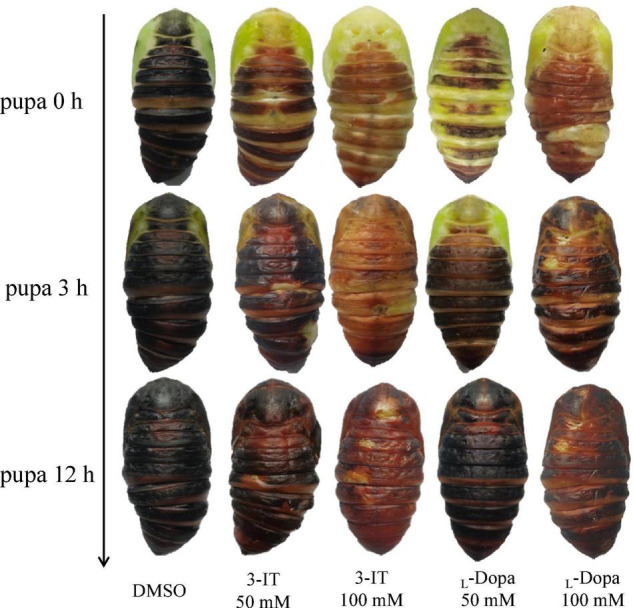
Color changes under different titers of 3-IT and L-DOPA during 12 h.

After 3-IT and _L_-DOPA injection, the proportion of healthy pupae decreased compared with the DMSO control group. The deformity rate of the control group was 10% after 30 μL DMSO injection, without any deaths, and the proportion of healthy pupae was 90%. We found that 50 mmol/L L-DOPA induced 16.7% deformity and 3.3% mortality; 100 mmol/L L-DOPA increased the deformity rate to 40% and mortality to 33.3%. The proportion of healthy pupae was 27.3%. In the 200 mmol/L L-DOPA group, the proportion of healthy pupae decreased to 10%, 66.7% of pupae died, and 23.3% exhibited deformities. Under the same chemical concentration of L-DOPA and 50, 100, and 200 mmol/L 3-IT, the 3-IT group showed deformity rates of 6.7, 30, and 16.7%, and mortality rates of 20, 36.7, and 66.7%, respectively. The proportion of healthy pupae under the same conditions was 73.3, 33.3, and 16.6%, respectively (Table 2). From the proportion of healthy and deformed pupae, we concluded that enzyme inhibitors influenced melanization and body color partially changed to brown or black. The higher concentration of inhibitors resulted in completely brown pupae and the influence of 3-IT outperformed that of L-DOPA (Figure 7).

**TABLE 2:** Statistics of pupae obtained after injecting pre-pupae of *A. pernyi* with *TH* and *DDC* inhibitors.

Inhibitors	Concentration (mmol/L)	Injection volume (μ L)	Total number of pre-pupa	Deformity rate (%)	Mortality rate (%)	Proportion of healthy pupae (%)
DMSO	10	30	30	10	0	90
L-α-methyl-DOPA	50	30	30	16.7	3.3	80
	100	30	30	40	33.3	27.3
	200	30	30	23.3	66.7	10
3-IT	50	30	30	6.7	20	73.3
	100	30	30	30	36.7	33.3
	200	30	30	16.7	66.7	16.6

### Inhibit Tyrosine Hydroxylase and DOPA Decarboxylase Will Influence Some Monoamines Variation

**FIGURE 8:**
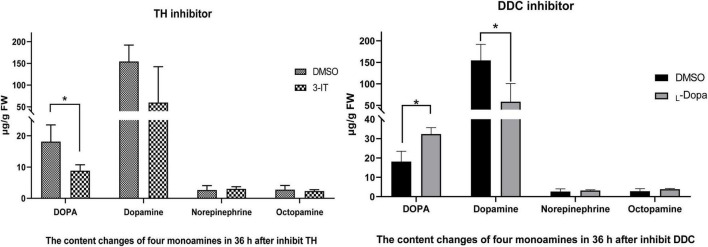
Content changes of four monoamines (including DOPA, Dopamine, Norepinephrine, and Octopamine) after TH and DDC enzyme inhibitors injection. Color changes under different titers of 3-IT and L-DOPA during 12 h. Three replicates were considered; the bar represents mean ± SD (*n* = 3), and significant differences were determined using Student’s *t*-test (*p < 0.05).

In 3-IT group, DOPA (8.85 μg/g FW, FW means fresh weight of sample) and Dopamine (60.02 μg/g) showed down-regulated compared with DMSO group (18.12 μg/g and 154.67 μg/g) at 72 h (36 h after inhibitor injection). DOPA in two groups showed significant difference (*p* < 0.05) while Dopamine do not show significant difference. Norepinephrine has little changes (3.06 μg/g) compared with control group (2.65 μg/g). Octopamine also shows very low content compared with DOPA and Dopamine, the content were 2.32 μg/g and 2.81 μg/g. After inhibit DDC enzyme with L-DOPA, Dopamine was down-regulated (58.72 μg/g) compared with control group (154.67 μg/g) (shows significant difference, *p* < 0.05). DOPA in treated group (32.33 μg/g) was higher than that of the control group (18.12 μg/g) (shows significant difference, *p* < 0.05). Other two monoamines, Norepinephrine and Octopamin, both have low content level (Figure 8).

## Discussion

The process of pupal emergence always lasts within 10 min in *A. pernyi*. Newly emerged pupae typically complete melanization in 24 h. To test melanin precursors involved in pupal melanization, we analyzed the time-course expression patterns of *TH* and *DDC*. Compared with previous research, more sampling time points were chosen for morphological and molecular characterization (Dai et al., 2015; Zhang et al., 2021). The expression patterns showed that mRNAs of both precursors were upregulated before pupal emergence, not during pupal melanism. RNAi and enzyme inhibitor experiments indicated that interfering with both mRNA and protein levels of TH and DDC will prevent pupal melanization.

Melanin is found not only in insects but is a ubiquitous pigment across the animal kingdom. Melanin is involved in the formation of adaptive color patterns, which can protect against ultraviolet radiation. Melanin is also involved in the immune response to pathogens or parasitoids (Dubovskiy et al., 2013). Melanization is common during development, although it varies among different insect species (Liu et al., 2015b). Silkworm pupae are always covered by cocoons and, therefore, the internal process of pupal formation cannot be observed easily. Under natural conditions, the pupae of the two most commonly reared silkworms, *B. mori* and *A. pernyi*, are typically yellow and black, respectively. Notably, color patterning depends on the environment, crypsis, mate recognition, warning signals, or thermoregulation (Michie et al., 2010). In *A. pernyi*, pupal melanism may be due to high ultraviolet irradiation and low temperature under natural conditions.

Tyrosine hydroxylase converts tyrosine to DOPA whereas DDC converts DOPA to dopamine. Several insects serve as models for studying the mechanism of melanism in phenotypic variations (Liu et al., 2010; Van’T et al., 2010). The melanic insect strains often overproduce DOPA and dopamine, a small amount of which is channeled to synthesize yellow or colorless pigments (Liu et al., 2015b). In this study, we focused on the dopamine pathway in black *A. pernyi* pupa generation under natural conditions. In the initial stage, pupae are easily wounded and prone to diseases due to the soft cuticle. Consequently, biological features drive insects to adapt to situations and change pupal conditions quickly for the next developmental stage (Koch, 2003). Different melanin pigmentation levels on the body surface could help them adapt their color according to the environment.

For this study, thousands of silkworms in the same cocooning stage were selected and analyzed. To ensure the selection of pre-pupae in the desired developmental stage and synchronous pupal emergence, the morphology of each pre-pupa was photographed during preliminary experiments. Based on these results, characteristic morphological changes and key gene expression patterns were observed at 60 h before pupal emergence.

ApTH and ApDDC amino acid sequences share high sequence homology with those of other insects. Diverse temporal expression patterns of TH in different insects may be related to species-specific regulatory elements in the upstream sequence (Gorman et al., 2007; Lee et al., 2015). In this study, both *ApTH* and *ApDDC* found many *cis*-element including CAAT-box. In tissue expression pattern, *ApTH* showed higher expression at 60 h compared with 24 h in epidermis and fat body. This coordinate with the result of qRT-PCR test in pupa epidermis. *ApDDC* has lower expression in epidermis at 24 h than midgut, while quickly up-regulated at 60 h which has more times change in midgut. In *B. mori*, *DDC* shows high level in the brain and ovaries/testes except epidermis (Wang et al., 2013). In the midgut of *A. pernyi*, we deduce the inner cuticle of gut in progress of cuticle construction or immune defense during pre-pupae stage.

In *B. mori*, *TH* is highly expressed during the late embryonic stage when the larval mouthpart ossifies *via* catecholamine synthesis (Liu et al., 2010). In this study, primarily from 48 to 72 h prior to pupation, high *ApTH* expression was detected. We deduce that *ApTH* and *ApDDC* are related to pupal melanism in *A. pernyi*. The main trigger of melanization does not seem to be the pupal emergence stage, but the altered expression of melanism-related genes, which anticipates the pupal stage by almost 2 days.

In north China, artificial methods [such as those reported by Wang et al. (2012)] are used to prevent pupal melanization in order to obtain attractive pupae which can be sold at a higher price. However, no strategy can prevent melanization if larvae have already undergone pupal changes. The cocoons must be placed in a suitable environment before pupation, with higher environmental temperature. This phenomenon and the molecular mechanisms may be also related with the Aspartate decarboxylase gene and β-alanine synthesis (data not published). The reverse phenomenon also can be found in black pupae (*bp*) mutant in *B. mori* (Dai et al., 2015). Indeed, the external temperature can influence body color in several insect species (Michie et al., 2010). The temperature may interfere with the melanin synthesis pathway, preventing DOPA melanin or dopamine melanin synthesis.

In this study, after injecting ds*TH* and ds*DDC*, some pupae did not show melanized cuticles. When injected with enzyme inhibitors, the color change was more evident. However, the inhibition of TH and DDC enzymes influenced the proportion of healthy pupae; results showed that a higher concentration of 3-IT and L-DOPA resulted in more deformities and increased mortality. A lack of these enzymes affected physiological processes and TH deficiency seemed to be more influential, as it resulted in a relatively higher mortality rate. RNAi of *TH* in mosquitoes, *Anopheles sinensis*, reportedly induced severe physiological defects during pupal metamorphosis (Qiao et al., 2016). CRISPR/Cas9 *TH* resulted in larval lethality in *Agrotis ipsilon* (Yang et al., 2018).

**FIGURE 9:**
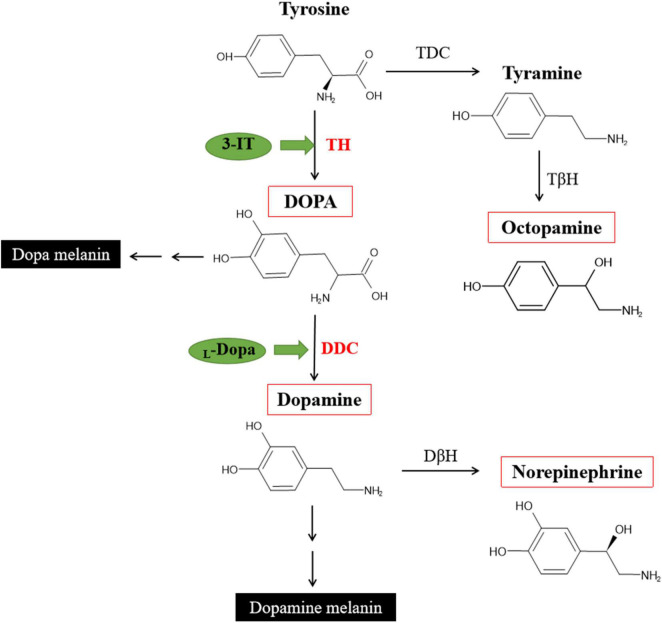
Partial pathways for cuticular melanization in insects and DOPA, Dopamine, Norepinephrine, Octopamine synthesis [modified from Wittkopp et al. (2003), Roeder (2005), and Lange (2009)].

In insects, the biogenic amines including Dopamin, Octopamine, Tyramine, Serotonin, and Histamine. Three of them were synthezed from Tyrosine (Wu et al., 2013). The DOPA melanin and Dopamine melanin synthesis are the reason of black or brown color in insects. In order to test influence TH and DDC will change the content of DOPA or Dopamine, not other monoamines, in the network of Tyrosine reaction. Four different monoamines were test in three different pathway (Figure 9). After inhibit TH, the DOPA showed lower content, and also in Dopamine (no significant difference). Inhibit DDC, the Dopamine was significant down-regulated while DOPA accelerated because of the transformation process interruption. Octopamine and Norepinephrine both showed no significant changes.

While we have found that *TH* and *DDC* upregulation started at the pre-pupae stage, the expression of these two genes was relatively lower at the beginning of pupal emergence. Although an entirely synchronous sampling at the pre-pupal stage is complex, the exact developmental process of each pupa after pupal emergence can be recorded and yield more significant data following the pre-pupal stage. Furthermore, other genes related to melanin synthesis might work, warranting further investigation.

## Conclusion

We identified two new genes related to melanin synthesis. After cocoon formation, the morphology of pupae was recorded by digital cameras from the pre-pupal stage to pupal melanization. During this process, the expression patterns of *TH* and *DDC* revealed the highest expression at 60 h (16 h before pupal emergence). RNAi of *TH* and *DDC* induced partial melanism in some pupae and enzyme inhibitors prevented melanism, even resulting in completely brown pupae. DOPA and Dopamine content will influenced by TH and DDC inhibitor. This research will help us understand the molecular mechanism during the pre-pupa to pupa transition in *A. pernyi*.

## Data Availability Statement

The datasets presented in this study can be found in online repositories. The names of the repository/repositories and accession number(s) can be found in the article/Supplementary Material.

## Author Contributions

YW and LQ: conceptualization and funding acquisition. QW and SZ: methodology. YB: software. JD and YB: validation. QW and WL: formal analysis. LZ and SZ: investigation. LZ and RY: resources. QW: data curation. YW: writing—original draft preparation and project administration. YW and QW: writing—review and editing. RY and JD: visualization. LQ: supervision. All authors have read and agreed to the published version of the manuscript.

## Conflict of Interest

The authors declare that the research was conducted in the absence of any commercial or financial relationships that could be construed as a potential conflict of interest.

## Publisher’s Note

All claims expressed in this article are solely those of the authors and do not necessarily represent those of their affiliated organizations, or those of the publisher, the editors and the reviewers. Any product that may be evaluated in this article, or claim that may be made by its manufacturer, is not guaranteed or endorsed by the publisher.

## Footnotes

1
https://www.expasy.org/

2
http://www.ncbi.nlm.nih.gov/Structure/cdd/wrpsb.cgi/

3
https://itol.embl.de/

4
http://bioinformatics.psb.ugent.be/webtools/plantcare/html/

## Supplementary Material

Supplementary Figure 1 Complementary cDNA and deduced amino acid sequence of ApTH. The initiation and termination codons, ATG and TAA, respectively, are indicated in bold; TAA is marked with an asterisk **(A)**. Exons in ApTH gene protein-coding region in DNA sequence **(B)**.

Supplementary Figure 2 Complementary sequence and predicted amino acid sequence of ApDDC **(A)**, and Exons in ApDDC gene protein-coding region in DNA sequence **(B)**.

Supplementary Figure 3 *Cis*-element prediction in genome sequence of *ApTH* **(A)** and *ApDDC* **(B)**, both of them including CAAT-box.
