# Supplementary figures and images for: Tyrosine Hydroxylase and DOPA Decarboxylase Are Associated With Pupal Melanization During Larval–Pupal Transformation in Antheraea pernyi
Source: Front Physiol. 2022 Apr 7;13:832730. doi: 10.3389/fphys.2022.832730 (PMC9022030; doi:10.3389/fphys.2022.832730)

## Slide 1
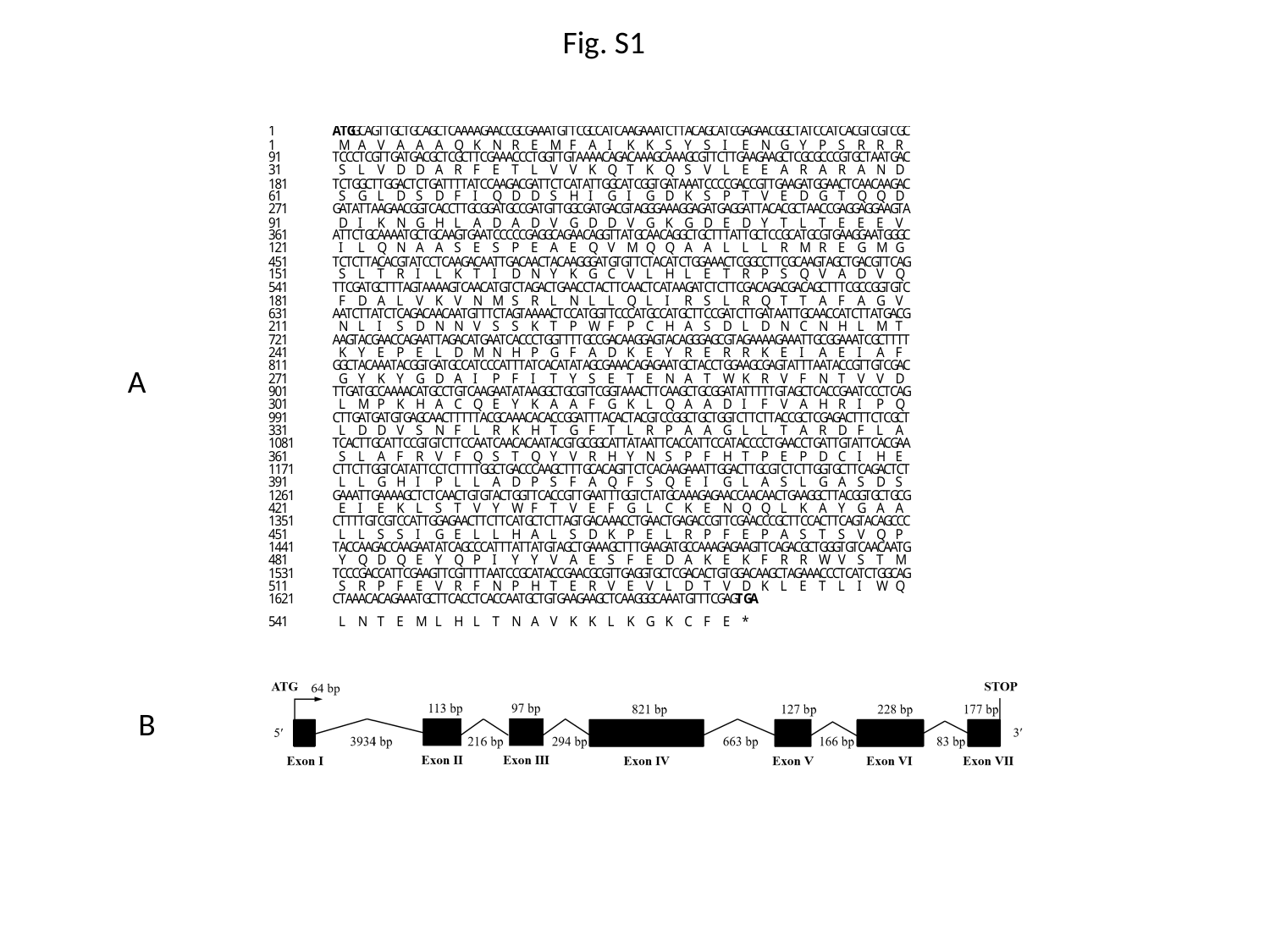

Fig. S1
A
B

## Slide 2
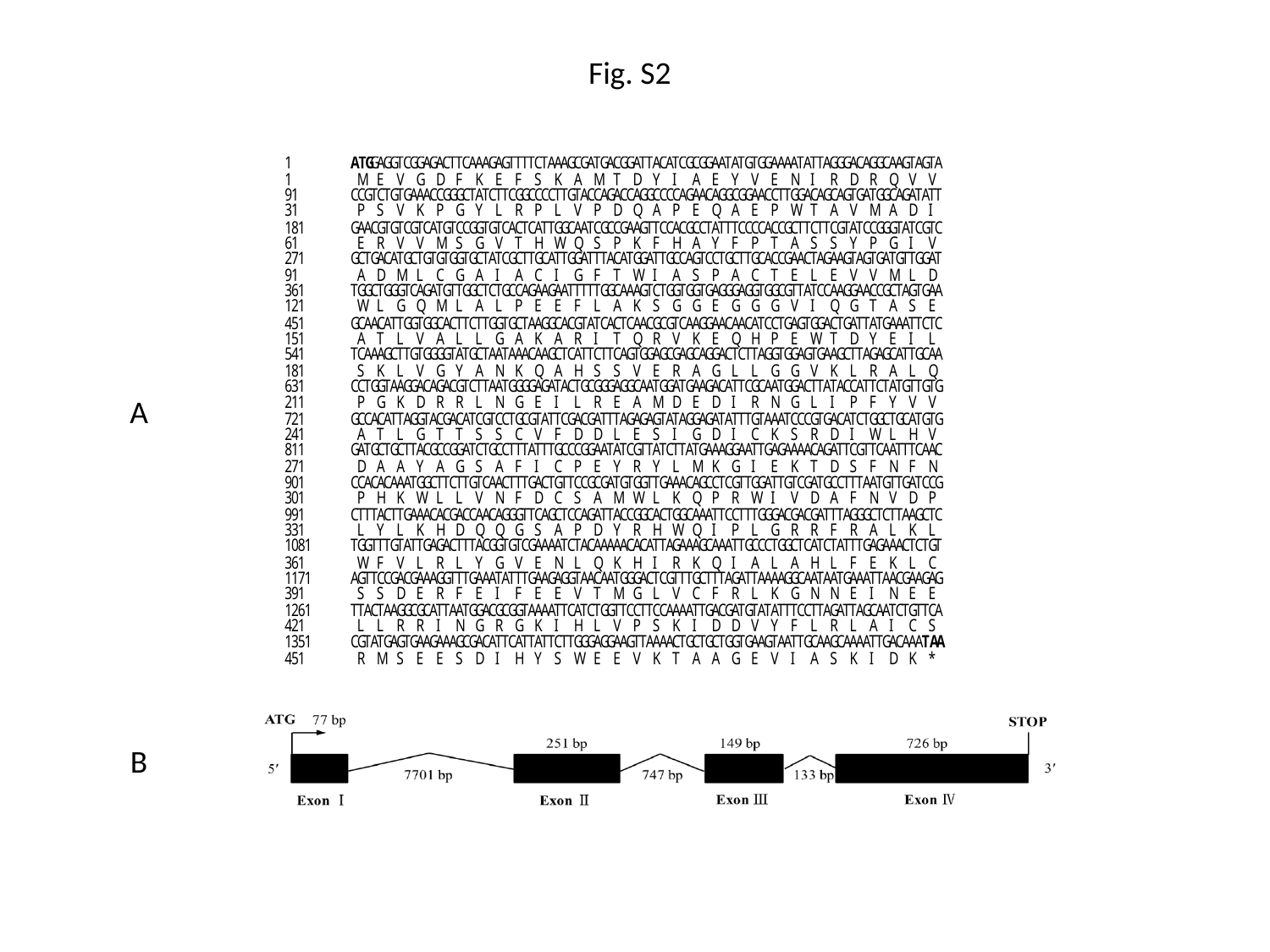

Fig. S2
A
B

## Slide 3
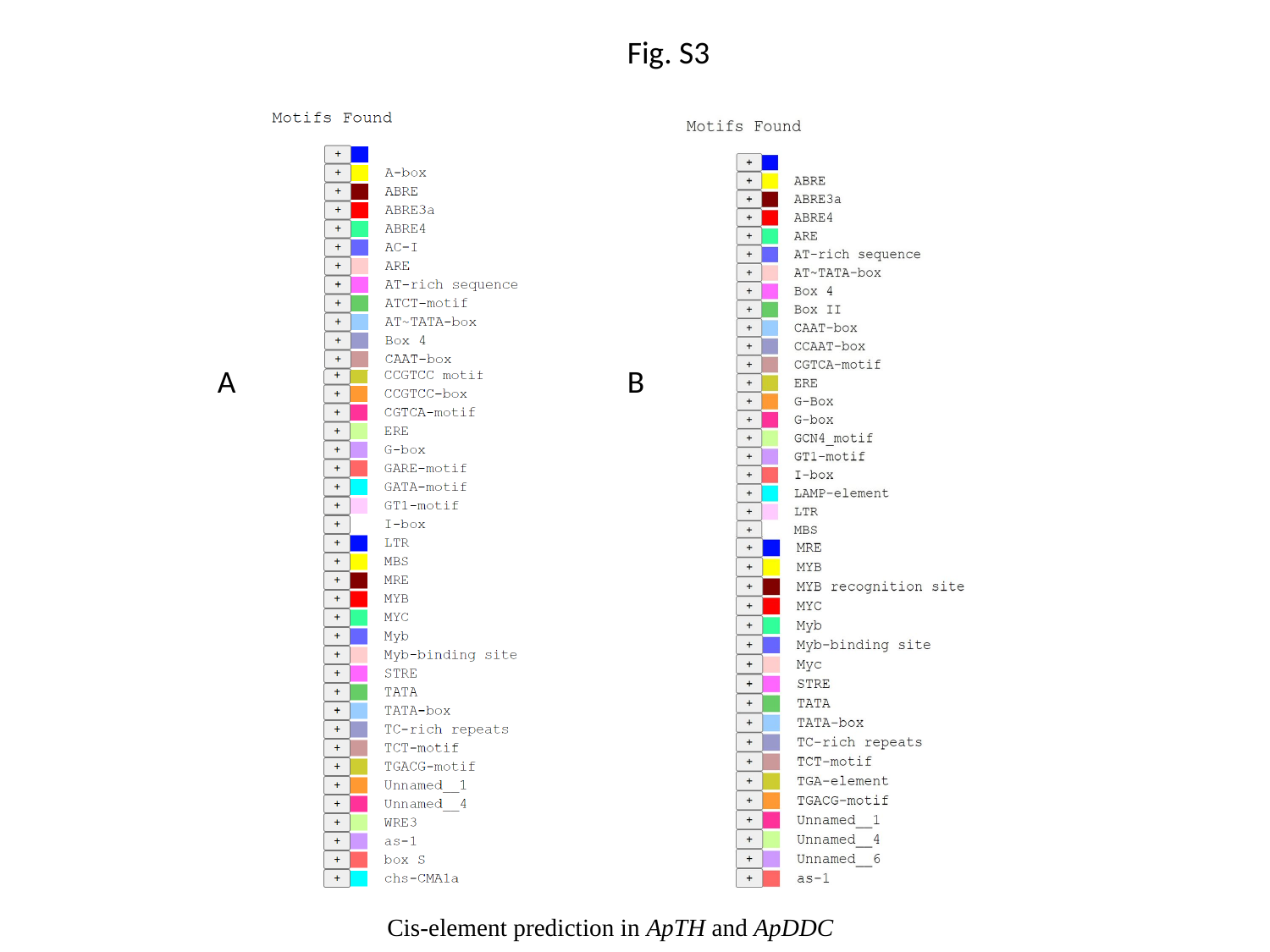

Fig. S3
A
B
Cis-element prediction in ApTH and ApDDC

Supplement: Supplementary Figure 1 — Complementary cDNA and deduced amino acid sequence of ApTH. The initiation and termination codons, ATG and TAA, respectively, are indicated in bold; TAA is marked with an asterisk (A). Exons in ApTH gene protein-coding region in DNA sequence (B). [file Presentation_1.pptx]
